# Occupational Risk Factors for Musculoskeletal Disorders among Workers in Dairy Diversification

**DOI:** 10.3390/healthcare12020178

**Published:** 2024-01-11

**Authors:** Fabien Buisseret, Nicolas Draye, Camille Di Santo, Jocelyn Pacewicz, Johanna Pannetier, Frédéric Dierick, Frédéric Telliez

**Affiliations:** 1 Laboratoire Forme et Fonctionnement Humain, CeREF, Rue Trieu Kaisin 136, 6061 Montignies-sur-Sambre, Belgium; drayen@helha.be (N.D.); la199486@student.helha.be (C.D.S.); la197565@student.helha.be (J.P.); 2 Service de Physique Nucléaire et Subnucléaire, Université de Mons, UMONS Research Institute for Complex Systems, 20 Place du Parc, 7000 Mons, Belgium; 3 MWSV—PreventAgri, Rue du Rabiseau 6, 6220 Fleurus, Belgium; johanna.pannetier@preventagri.be; 4 Centre National de Rééducation Fonctionnelle et de Réadaptation—Rehazenter, Laboratoire d’Analyse du Mouvement et de la Posture (LAMP), 1 Rue André Vésale, 2674 Luxembourg, Luxembourg; frederic.dierick@rehazenter.lu; 5 Faculté des Sciences de la Motricité, UCLouvain, 1-2 Place Pierre de Coubertin, 1348 Ottignies-Louvain-la-Neuve, Belgium; 6 Laboratoire Péritox UMR_I 01, Institut d’Ingénierie de la Santé-UFR de Médecine, Université de Picardie Jules Verne, Rue des Louvels, 80036 Amiens, France; frederic.telliez@u-picardie.fr

**Keywords:** dairy products, farming diversification, musculoskeletal disorders, pain, risk analysis

## Abstract

Background: In a changing European agricultural context, diversification of dairy farms is gaining attention. This study seeks to (1) assess musculoskeletal pain prevalence associated with tasks such as butter, yogurt, and cheese production; and (2) analyze associated risks. Methods: Observing 31, mostly female, workers, we utilized the ERGOROM questionnaire, a methodology adapted from the Institut National de Recherche et de Sécurité, and Key Indicator Method forms. Results: Findings revealed that tasks like load carrying (42% of workers), manual work (17%), and awkward postures (14%) resulted in musculoskeletal pain, predominantly in the lower back (65%), neck (39%), and dominant upper limb areas (shoulder: 61%, elbow: 26%, and wrist: 65%). While psychosocial risks remained low, concerns arose from workload, hygiene standards, and resource unpredictability. Conclusions: As dairy farming evolves from artisanal to semi-industrial, our study emphasizes the importance of ergonomic adaptations to protect farmers’ health and prevent musculoskeletal disorders during diversification.

## 1. Introduction

Diversification in agriculture, characterized by farmers engaging in non-agricultural economic activities using their existing facilities, is becoming increasingly common among European farmers [1]. For example, the number of farms engaging in direct sales of their products increased by 12.3% between 2016 and 2020 in Belgium [2]. The process of farm diversification can be segmented into two distinct categories: diversification in production modes and types, and diversification through the integration of non-agricultural activities linked to the farm. This study focuses exclusively on the latter category, encompassing endeavors like food production, agritourism, and educational initiatives held on the farm. Of particular interest within this context is the diversification of dairy farms, specifically the on-farm production of butter, yogurt, or cheese using locally produced milk. The authors of [3] have shown the potential of such value-added diversification in fostering resilient pathways of economic development for small dairy farms.

Recent studies in the field of occupational health have increasingly underscored the significance of musculoskeletal disorders (MSD) in agricultural settings, particularly within the dairy farming sector. A comprehensive systematic review elucidated various risk factors contributing to MSD among farm owners and workers, highlighting critical elements such as prolonged duration of farming activities, extensive hours spent in animal barns, and the physical demands of milking large herds [4]. Notably, the low-back emerged as the most affected region, followed by the lower and upper extremities, underscoring the physical toll of farming activities on these body parts. Also, according to the systematic review by [5], the most common MSD among farmers mainly affect the back, neck, and upper extremities. These findings are supported by additional research, which confirms that MSD are a predominant hazard in agricultural occupations, especially in roles involving significant physical labor. The repercussions of these disorders extend beyond personal health, manifesting as increased disability rates, loss of work time, and elevated production costs, thereby impacting the agricultural sector’s overall efficiency and sustainability [6].

The ergonomic aspects of modern dairy farming practices have also been scrutinized, with a focus on the historical evolution of these practices and their corresponding ergonomic risks [7]. This review has revealed a notable gap in research, particularly concerning the direct measurement of exposure to risk factors among dairy farm workers using ergonomic assessment tools [7]. The physical demands of dairy farming practices, including cow milking, have been linked to MSD affecting the knees. Furthermore, epidemiological studies across various national contexts have consistently reported the prevalence of musculoskeletal symptoms and disorders among dairy farm milkers. These findings indicate the pervasive nature of MSD in the dairy industry, transcending different production systems and geographical locations. Despite this, a comprehensive understanding of the high risk of MSD in dairy production remains elusive [8]. This is echoed in a study conducted in Sweden, which investigated and compared work-related MSD among dairy farmers and employed farm workers, highlighting the physically demanding nature of dairy farming and its strong association with musculoskeletal discomfort [9]. Moreover, farmers often exhibit a lower propensity to seek medical advice compared to other occupational groups [10], thereby increasing the risk of chronic pain, and worsening of health within this population. As farm diversification necessitates a departure from conventional farming activities, a question arises: does the adoption of diversification introduce specific or additional MSD risks among farmers? In the context of dairy farm diversification, tasks inherently involve risk factors such as carrying heavy loads (>20 kg), repeated gestures (mixing ingredients, packaging, etc.) or awkward postures induced by space constraints in workshops.

The objectives of the study are: (1) to conduct a comprehensive descriptive analysis of the target population, elucidating prevalent musculoskeletal pain experienced; and (2) to carry out an in-depth risk analysis of tasks undertaken by farmers engaging in dairy diversification.

## 2. Materials and Methods

### 2.1. Population

To qualify for participation in this study, farms needed to be in Wallonia, the French-speaking region of Belgium, and to specialize in milk production. In addition, it was essential that these farms process their milk on-site into products like butter, yogurt, or cheese. The recruitment of farms was conducted through e-mail and telephone contacts, leveraging both personal networks of the authors and connections from PreventAgri, a Walloon organization focused on safety and prevention in agriculture and related green sectors.

The sole criterion for an individual’s participation in the study was to be a worker of legal age actively involved in the dairy processing activities on the enrolled farm. The study excluded individuals who declined to participate, those engaged in activities conductive to a thorough risk assessment using our selected ergonomic methods, or workers with visible motor disorders or diagnosed neurological, neuromuscular, degenerative, or rheumatic conditions that might affect their perceived or observable work constraints.

### 2.2. Protocol

The research protocol received approval from the Comité Académique de Bioéthique under file number B200-2022-107. Each participating worker was initially administered the 40-item ERGOROM questionnaire [11], based on the Nordic questionnaire [12]. The ERGOROM questionnaire, comprising 40 items, retains the demographic section and the upper limb + lower back analysis of the Nordic questionnaire, making the two tools broadly similar. However, ERGOROM is more concise. It omits the lower limb analysis, which was deemed less relevant based on initial risk assessments of tasks in dairy milk diversification. Unique to ERGOROM is a section dedicated to evaluating psychosocial risks. Furthermore, while ERGOROM records instances of pain experienced in the past 12 months, the Nordic questionnaire provides a more comprehensive assessment, detailing the number of days pain was experienced within the same time frame. The ERGOROM questionnaire consists of four sections: (1) A demographic section, which collects information on the respondent’s personal characteristics such as age, weight, height, gender, laterality (the dominant limb was the one used to write), smoking status and extra-professional hobbies involving physical effort; (2) A work section, which assesses past and current working conditions; (3) A section on MSD symptoms, with questions on any pain experienced over the past 12 months in the neck, shoulders, elbows, wrists and lower back; (4) A section on perception and appreciation of working conditions. Regarding complaints, ERGOROM mainly assesses complaints and tasks relating to the upper limbs and back, as these are prominent in the agricultural milieu [5]. In the fourth section, the ERGOROM questionnaire also incorporates five items grounded in Karasek’s job demands–control model [13,14] for the assessment of psychosocial risks: (1) “Are you satisfied with your relations with your colleagues?”; (2) “Are you satisfied with your relations with your superiors?”; (3) “Are you satisfied with your current job?”; (4) “Do you feel appreciated in your work?”; (5) “Do you feel tense, uptight or stressed?”. These items are each scored between 1 and 5, the lowest value being the worst level of satisfaction. The total score of these items generates an “ERGOROM psychosocial risk” composite score. After the questionnaire, an analysis of worker tasks was conducted simultaneously by two observers (Camille Di Santo and Jocelyn Pacewicz), whose conclusions were harmonized through consensus.

First, the workers’ subjective rankings of the relative physical demands associated with the three key tasks involved in final product production (butter, yogurt, or cheese) were solicited. From these, the two or three most physically demanding tasks reported by workers were selected for observation—not all workers were involved in at least three tasks.

Second, a comprehensive analysis of physical workload was undertaken on these tasks by resorting to an adapted version of the risk analysis of workload method by the “Institut National de Recherche et de Sécurité pour la prévention des accidents du travail et des maladies professionnelles” (INRS is the French National Research and Safety Institute for the prevention of occupational accidents and diseases) [15]. Items belongs to five categories: (1) physical efforts (7 + 4 items), (2) dimensioning (7 + 3 items), (3) temporal characteristics (7 items), (4) environmental characteristics (5 items), (5) organization and psycho-social aspects (7 + 2 items). The nine additional items are taken from the FIFARIM method, which focuses on manual handling tasks [16]. The following items have been added for their relevance in dairy milk diversification: (1) Physical effort: sustained effort with arms above mid-trunk, using the hand as a tool for striking or tearing, palmar grip (full hand) of loads >5 kg and digital grip (thumb-index) of loads >1 kg, prolonged static force of hands or fingers; (2) Dimensioning: visible neck flexion, tilt and rotation, wrist out of neutral position, highly bent elbow or rapid forearm rotation; (3) Organization and psycho-social aspects: recognition, good contact with colleagues. The assessment quantified the 42 items according to their severity, encompassing four ergonomic risk levels: minimal, acceptable, conditionally acceptable, and unacceptable. The minimal risk implies that the risk level is very low and may not require immediate intervention. This typically applies to situations where the work environment and tasks are designed in such a way that they pose almost no health or safety risk to the workers. The acceptable risk implies work conditions or risks that are considered safe for workers without posing significant health risks, and they comply with legal and best practice standards. The conditionally acceptable risk is used for situations where the risk is moderate but can be managed with certain conditions or improvements. It often implies that while the current state meets basic legal requirements, there is room for improvement to enhance safety and ergonomics. This might involve regular monitoring, specific measures to mitigate risks, or plans for future improvements. The unacceptable risk is applied to situations that pose a high risk to workers’ health and safety. These conditions do not meet legal or ergonomic standards and require immediate action to rectify the issues. Unacceptable conditions are those that are likely to cause harm or pose significant risks to workers, including potential violations of health and safety regulations. Several items in the INRS questionnaire correspond to quantifiable metrics, such as the distance of charge displacement and the number of repetitions per minutes. In contrast, other items are scored based on visual observation. These methodologies and their specifics are detailed in [15]

**Figure 1:**
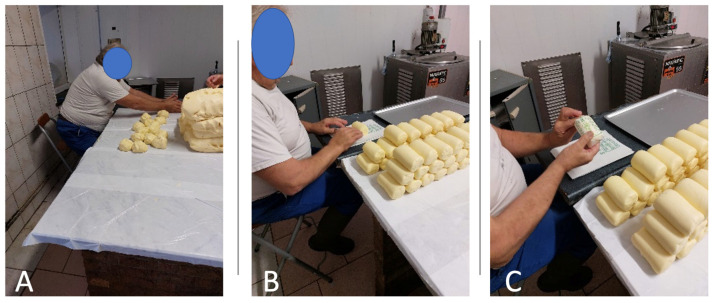
Example of a farmer forming butter clods (**A**) and packing them (**B**,**C**).

Third, the Key Indicator Method (KIM) was applied. KIM relies on objective indicators, like frequency and duration of movements, weight of loads handled, number of repetitions, etc. [17]. The observer must fill the most relevant KIM forms for a given task, from a range of six options available [18]: LHC (manual Lifting, Holding and Carrying loads), ABP (Awkward Body Postures), MHO (Manual Handling Operations), PP (Pushing/Pulling). Each task was analyzed utilizing the most appropriate KIM form. For example, the “butter packaging” task displayed in Figure 1C corresponded to the MHO KIM form, centered on “manual work”. The BF (Whole Body Forces) and BM (Body Movement) forms were deemed unsuitable for observed tasks. For each form, a KIM score is calculated. This score is derived from the multiplication of a time-rating factor by another factor that is based on various indicators such as posture, force, and frequency. These factors are determined from specific items within each form, which quantitatively represent observed behaviors. For more detailed information, including access to the KIM forms, readers are encouraged to consult reference [19].

Tasks were ranked based on their corresponding KIM scores. A moderate KIM score (between 20 and 50) indicates that the task being assessed is associated with a physical load that can be physically demanding but remains manageable with appropriate preventive measures and work planning. A KIM score between 50 and 100 indicates that the task assessed is associated with a physical load that may affect workers normally able to work under pressure and that preventive measures should be considered. A high KIM score (>100) indicates that the task being assessed is associated with a probable physical overload that could lead to MSD if work adjustment measures are not put in place.

### 2.3. Data Analysis

The results of the ERGOROM questionnaire, INRS and KIM methods are summarized using descriptive statistics in the form of summary tables and plots. Note that the raw data are available on the OSF online scientific data repository platform [19]. The Pearson correlation coefficient r was calculated between the ERGOROM psychosocial risk score and the average weekly working hours. An Analysis of Variance (ANOVA) was conducted on the KIM scores to assess task-related differences, using a significance level of 0.05. All statistical analyses were performed using R software (v. 4.3.1).

## 3. Results

### 3.1. Population

A total of 15 farms, meeting the established inclusion criteria, responded affirmatively to the call for participation and were consequently selected. Among these, eight produce butter, four yogurt and three cheese at the time of the observation. Four days a week over four weeks were allocated to farm visits in November 2022. Visiting a farm and processing the associated data took 1 day, and two observers (Camille Di Santo and Jocelyn Pacewicz) were always simultaneously present. An average of 6 h were spent observing and filling questionnaires in for each farm.

The study encompassed a cumulative total of 31 workers and 96 h of observation. Several observations were carried out, depending on the tasks performed by the workers: Each worker was asked to list the two or three most arduous tasks they had to perform regarding their professional activity in diversification. Twenty-seven workers listed two tasks, and four workers gave three tasks. This led to the completion of 66 KIM forms and 66 INRS forms in total. Observers spent an average of 6 h in each farm, i.e., approximately 3 h per worker and 1.5 h per task. An example of an observed work situation is shown in Figure 1.

The main characteristics of the workers are summarized in Table 1. Most workers were women, aged over 34, working more than 30 h (median) per week in diversification. This was their only professional activity. The workers worked for a median of 7 years in their current position. A notable observation emerges wherein 15 out of 31 workers were identified as members of the farm owner’s family. Worth highlighting is the consistent male representation among farm owners across all 15 farms. Workers presented a low psychosocial risk profile. A significant correlation (Pearson correlation coefficient = −0.45, *p* = 0.02) between the weekly working hours and the ERGOROM score for psychosocial risk was found.

**Figure 2:**
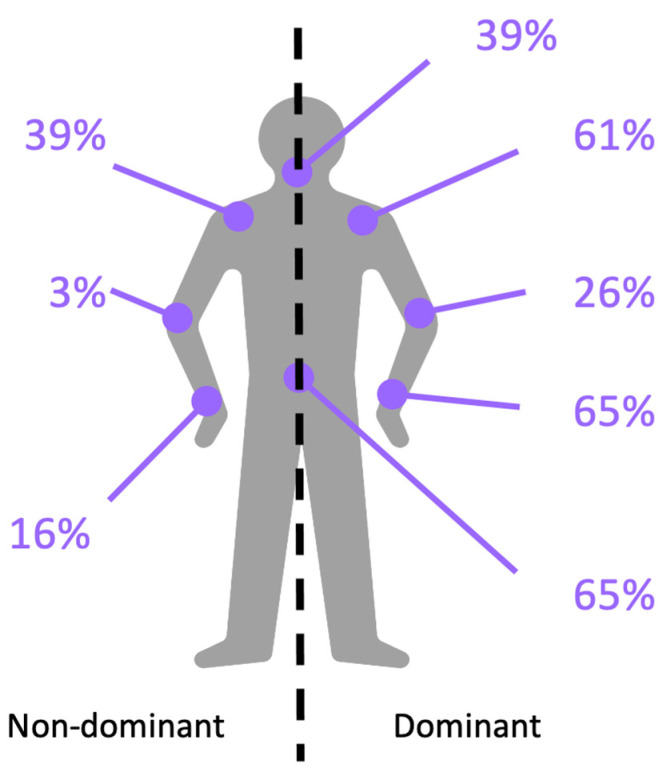
Percentage of workers reporting trunk and upper limb pain. The dotted line separates the dominant (D) and non-dominant (ND) limbs. Clockwise from ND wrist: ND elbow, ND shoulder, neck, D shoulder, D elbow, D wrist, lumbar region.

Regarding the location of reported pain represented schematically in Figure 2, it appears that a preponderance of pain localized in the dominant upper limb and the lumbar region is notable. The median number of painful areas reported is two.

Following the INRS analysis, it appears that 42% of workers identified load carrying as the most physically demanding aspect of their work. Manual work and awkward postures were respectively reported as the most physically demanding aspects of the job by 17% and 14% of workers.

### 3.2. Workplace Analysis

**Figure 3:**
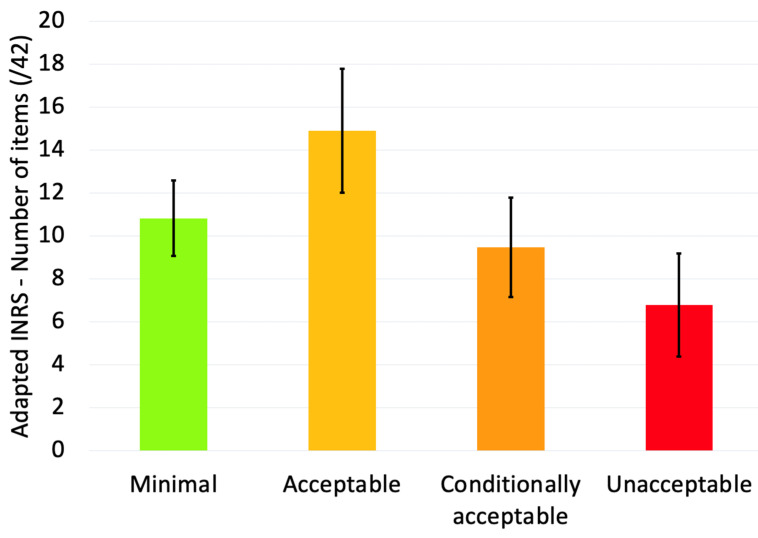
Average number of items (±standard deviation) associated with a minimal, acceptable, conditionally acceptable, or unacceptable risk by the INRS method in the workers observed. The 42 items belong to the following categories: (1) physical efforts (7 + 4 items), (2) dimensioning (7 + 3 items), (3) temporal characteristics (7 items), (4) environmental characteristics (5 items), (5) organization and psycho-social aspects (7 + 2 items).

**Figure 4:**
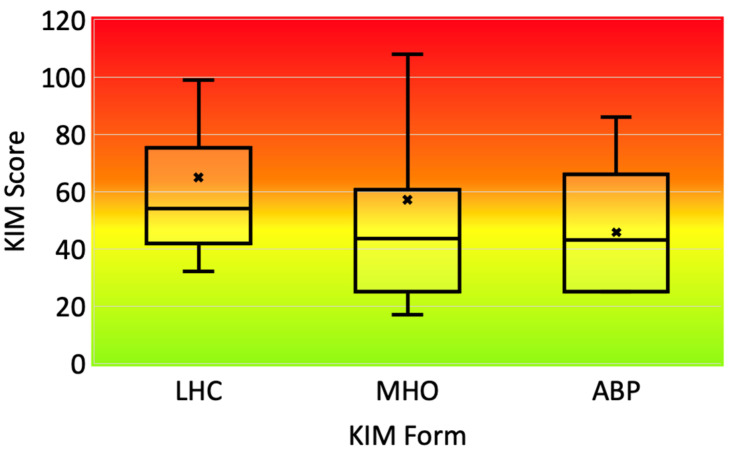
Boxplots summarizing the KIM scores calculated for the various filled forms. The color gradient highlights low-risk (green), intermediate-risk (yellow) and high-risk (red) areas for MSD. PP forms are not displayed since too few of them were filled. Abbreviations: KIM (Key Indicator Method), LHC (manual Lifting, Holding and Carrying loads), MHO (Manual Handling Operations), ABP (Awkward Body Postures). The crosses are the average scores for each form.

**Figure 5:**
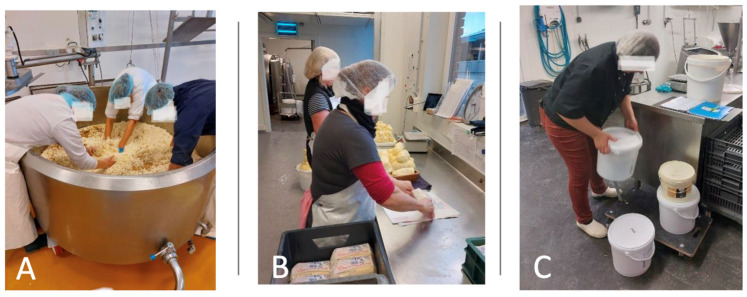
(**A**) Task related to KIM ABP form: workers in the churn. (**B**) Task related to KIM MHO form: packing butter clods. (**C**) Task related to KIM LHC form: handling buckets filled with yogurt.

The comprehensive analysis of the various tasks scored using the adapted INRS method is presented in Figure 3. The KIM scores calculated for each form are given in Table 2 and displayed in Figure 4. Visual observation indicates that tasks involving manual handling of loads (LHC) and manual work (MHO) yield the highest KIM scores. However, a non-parametric ANOVA revealed that the median scores between the tasks are not significantly different (*p* = 0.111). This non-parametric approach was chosen due to the failure to meet normality assumptions as indicated by the Shapiro–Wilk normality test (*p* < 0.001). Example of specific tasks related to different KIM forms are given in Figure 5.

## 4. Discussion

The historical management of farms has traditionally been dominated by males, while farm diversification has primarily been undertaken by farmers’ families, including their wives. This distribution of professional roles persists today. In 2020, a study conducted in Wallonia revealed that 85% of farm managers were male, while women comprised 29% of the regular workforce, mainly holding associate roles on Walloon farms [2]. In line with this finding, our study sample predominantly consisted of women, constituting 84% of the population (Table 2), of which 35% were identified as farm managers’ wives. Other workers were mainly employees, with 17 out of 21 being female, including some family members. We can therefore assume that our sample accurately represents the gender distribution in dairy farm diversification in Belgium. Diversification activities among the observed workers are notably significant, with a median reported time investment of 30 h. The main task observed among these activities was butter production.

Given that our population showcases a notable prevalence of women engaging in diversification, it raises the question of whether women are more susceptible to developing Musculoskeletal Disorders (MSD) than men. In [20] they used a modified standardized Nordic questionnaire and a rapid musculoskeletal assessment score sheet to collect data, as well as logistic regression methods to detect factors influencing MSD among 138 manual labor farmers in India. Given that the ERGOROM questionnaire is based on the Nordic questionnaire for upper limb analysis, it is feasible to reliably compare their results with ours. Results showed that, except for the shoulders, gender was the most significant factor influencing MSD across all upper body regions. The type of solicitations and constraints in occupational as in private life of women vs. men could be at the origin of this result, although it remains to be elucidated.

As previously mentioned, 65% of the surveyed workers reported experiencing pain in the lumbar region, often attributed to load carrying. Several studies, such as [21], confirm the relationship between low-back pain and load bearing among 600 Irish farmers. The results of the study showed that among the 38 farmers with low-back pain due to farming activity, the majority targeted load carrying as the source of injury, followed by repetitive work. Global flexion of the spine could also be associated with the low-back pain described by 65% of farmers. The systematic review [22] established that awkward working postures, including stooping and squatting, correlate with an increased risk of low-back disorders among farmers. According to [23], out of 1130 workers (792 men, 338 women) in the agricultural sector (not only farm diversification), the highest prevalence, over the last 12 months concerned the shoulder in men (37%) and the hand/wrist area in women (49%). These two areas are also among the most painful in our workers. In our study, lower back (65%), neck (39%) and, on the dominant side, shoulder (61%) and wrist (23%) emerged as the most painful regions, aligning with the findings of [9] in Sweden.

Psychosocial factors play an important role in the onset and/or duration of MSD. Indeed, work-related MSD originate not only from physical stressors but also from psychosocial risks like job stress and dissatisfaction [24]. In this last work it is also suggested that, once an injury has occurred, psychosocial factors such as depression and inappropriate reactions to pain play a central role in the transition from acute to chronic pain, and in the development of disability. Our assessment revealed low levels of psychosocial risks within dairy diversification (Figure 3). Psychosocial factors seem rather positive in the context of diversification activity and could have a protective effect/represent health-promoting factors on the occurrence of MSD. Stressors identified in worker discussions included weekly workload, fear of not being able to meet demand, compliance with hygiene standards by the Federal Agency for the Safety of the Food Chain (AFSCA), and uncertainties about staff availability or machine operation. However, broader societal contexts were not considered in our questionnaires, such as the current energy crisis, for example. In this regard, it is worth noticing that, according to [25], small farms involved in diversification were more resilient than traditional small farms during the COVID-19 pandemic, suggesting that this business model must be encouraged among farmers.

Load carrying emerges as the most challenging task, involving lifting and moving heavy containers of milk, cream, and butter (see Figure 5C), some weighing up to 20 kg. Lifting is generally performed with lumbar flexion, as the containers are mostly stored on the ground; the loads must be placed precisely on the workshop table, which is an intense effort. This type of constraint in milk production has already been quantified among farmers in Brazil [26]. The perceived difficulty of these load-bearing tasks is objectified by KIM analysis, which yields the highest average score (Figure 4), partly due to our predominantly female subject group and the KIM method, which, being based on the ISO 11228-1 standard, differentiates between genders [27].

INRS analysis reveals a significant risk associated with awkward postures. The repetitive nature of certain frequently observed manual tasks, like butter packaging and sizing (Figure 5B), maintained for several hours with the head bent and arms slightly raised, is a high-risk factor for MSD, and correlates with the pain reported in the lumbar and cervical regions, for example. Even if the INRS analysis globally reveals an acceptable risk level, the potential strain of seemingly “light” tasks in the long term must be considered [28]. Another way of assessing the risk of developing MSD would be to analyze a dairy-processing worker over a full week, considering all the tasks performed in the production of all milk-based products. This analysis will be addressed in future research.

Biomechanical strategies and psychosocial factors are intertwined: there is indeed a complex relationship between the response to physiological stress and chronic pain symptoms [29]. This relationship has been demonstrated (observed) in supermarket cashiers [30]. The significance of studying awkward postures and their connection to MSD extends well beyond the realm of farming. For instance, a systematic review [31], which analyzes the risk factors associated with MSD in the workplace, identifies awkward postures and excessive repetition as notable biomechanical risk factors for the development of MSD in the elbow or forearm. However, another systematic review concludes that there is no association between awkward postures and low-back pain [32]. Consequently, while some associations with MSD are evident, the impact of awkward postures on low-back pain is not supported, highlighting the complexity and ongoing evolution of research in this field.

Our study has several limitations. Firstly, the specific dairy product observed varied depending on the day of the visit, which was dictated by each farm’s unique weekly production schedule. While our focus was primarily on butter production, a week-long observation across different farms could have provided a more comprehensive insight into the diversity of products. Secondly, our assessments did not account for the frequency of tasks performed over different time spans (weekly, monthly, yearly), which is crucial in determining the risk of a workstation, even for tasks not performed frequently. Thirdly, our questionnaires were solely focused on the workers’ current musculoskeletal pain related to their diversification work tasks. Including questions about musculoskeletal pain related to past work would have been valuable in assessing the specific impact of diversification. Lastly, the interviews were conducted in the workplace, where many were family members. This setting may have influenced some workers to underreport issues, particularly those related to psycho-social risks.

## 5. Conclusions

Our study among workers in dairy farm diversification indicates that manual handling risk emerges as a major constraint through KIM assessment. This risk factor potentially contributes to the high prevalence of lumbar complaints. Immediate adjustments to workspaces and equipment are generally feasible and adapting the workstation is an essential first step. In [19], we proposed pedagogical documents containing guidelines for workers in dairy diversification based on our present risk analysis and on the active participation of the workers. We emphasized the feasibility of adapting the work around the churn: using horizontal rather than vertical churns, using carts with adaptable heights, etc. We also introduced resource movements to reduce or prevent pain so that workers may adapt their movements to improve mobility and strength. The dissemination of these guidelines is currently included in training courses given by PreventAgri for the Green Sectors in Wallonia. In Belgium, most farmers are self-employed, which means they are not subject to regular medical surveillance. That is why such training courses are of particular importance.

The shift from artisanal to semi-industrial dairy diversification, without ergonomic modification/adjustments, would greatly increase the risk of MSD due to intensified exposure to constraints. For example, the ABP KIM form has an average score of 46 for an average time factor equal to 1.3 h per day. If a worker specializes in tasks with awkward postures, the time factor will increase. In this case, we estimate that a time factor of 3 h per day would lead to a high-risk situation, with a KIM score over 100. While the role of ergonomists is vital in mitigating prolonged or repetitive work in awkward postures, this support remains relatively underrepresented, at least in Belgium. The growing importance of diversification in agricultural activities should, however, be followed by an increase in the resources allocated to this type of prevention, to safeguard farmers’ health.

## Figures and Tables

**Table 1:** Characteristics of workers included in the study. Results are expressed under the form median [1st quartile–3rd quartile].

N	31
Sex (M/F)	5/26
Handedness (Left-handed/Right-handed)	4/27
Age (years)	45 [34–54]
Weight (kg)	72 [60–81]
Height (cm)	165 [160–172]
Workload in diversification (hours/week)	30 [24–34]
Experience in diversification (years)	7 [2–16]
ERGOROM psychosocial risk (/25)	23 [22–24]

**Table 2:** KIM scores calculated for the various filled forms. Results are expressed under the form median [1st quartile–3rd quartile]. Only three PP forms were filled, hence the individual values are given in this case. A KIM score between 50 and 100 indicates that preventive measures should be considered. A KIM above 100 indicates that the task could lead to MSD if work adjustment measures are not put in place. Abbreviations: KIM (Key Indicator Method), LHC (manual Lifting, Holding and Carrying loads), MHO (Manual Handling Operations), PP (Pushing/Pulling), ABP (Awkward Body Postures).

KIM Form	Number	Score
PP	3	17, 22, 42
ABP	15	43 [28–55]
MHO	29	44 [27–60]
LHC	19	54 [44–74]

## Data Availability

Data are available in the OSF deposit https://doi.org/10.17605/OSF.IO/WXES8. English translations of our questionnaires are also available.
